# Cutaneous involvement as the presenting sign of monomorphic/epitheliotropic intestinal T-cell lymphoma (MEITL): A case report

**DOI:** 10.1016/j.jdcr.2025.07.035

**Published:** 2025-09-05

**Authors:** Karin Warshavsky, Shir Toubiana, Ginette Schiby, Aviv Barzilai

**Affiliations:** a Dermatology Department, Sheba Medical Center, Ramat-Gan, Israel; b Gray Faculty of Medical and Health Science, Tel-Aviv University, Tel Aviv, Israel; c Pathology Department, Sheba Medical Center, Ramat-Gan, Israel

**Keywords:** dermatopathology, lymphoma, MEITL, oncodermatology, T-cell Lymphoma

## Introduction

Monomorphic epitheliotropic intestinal T-cell lymphoma (MEITL) is a rare and aggressive T-cell lymphoma primarily affecting the gastrointestinal (GI) tract but can also spread to extra-intestinal sites like the lymph nodes, lungs, and central nervous system. Skin involvement is exceedingly rare, with only a few cases reported in the literature.^1^ Here, we present a case of MEITL that initially manifested in the skin, posing a significant diagnostic challenge.

## Case report

A 49-year-old male was referred to the dermatology department due to the gradual appearance of several new, asymptomatic plaques and nodules, that progressively increased in number. The patient reported persistent abdominal pain and dyspepsia; however he denied any night sweats or weight loss. He had no relevant medical history. The patient's family history was positive for Celiac disease (CD), though his serology for CD was negative.

Two months prior to the referral, the patient experienced abdominal pain. Abdominal ultrasound suggested terminal ileitis, but endoscopy showed no GI abnormalities. Duodenal biopsy revealed preserved villi architecture with mild increase in intraepithelial lymphocytes.

**Fig 1:**
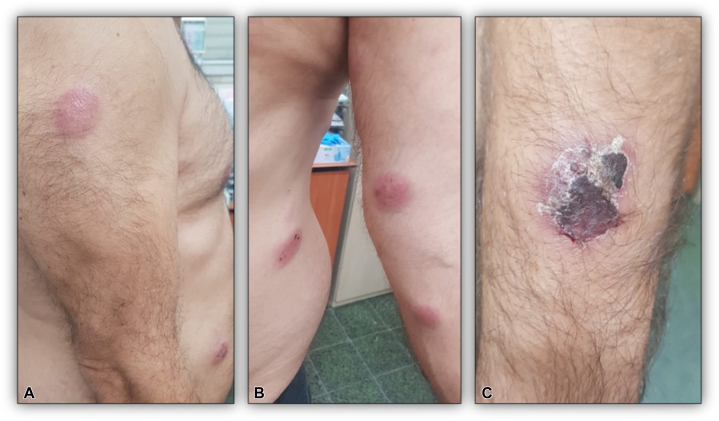
**A** and **B,** Disseminated erythematous plaques and nodules and **(C)** some show scaling and hemorrhagic crust.

Skin examination revealed an asymmetric rash over the trunk, arms, and thighs, composed of scattered erythematous plaques and nodules, few of them showing scaling and hemorrhagic crust (Fig 1). The rest of the physical exam was unremarkable, with no evidence of clinical lymphadenopathy.

Laboratory tests revealed normocytic anemia and elevated lactate dehydrogenase (LDH) levels (315 U/L). HIV, hepatitis, and HTLV-1 serologies were negative, while Epstein-Barr virus (EBV) serology showed past infection.

**Fig 2:**
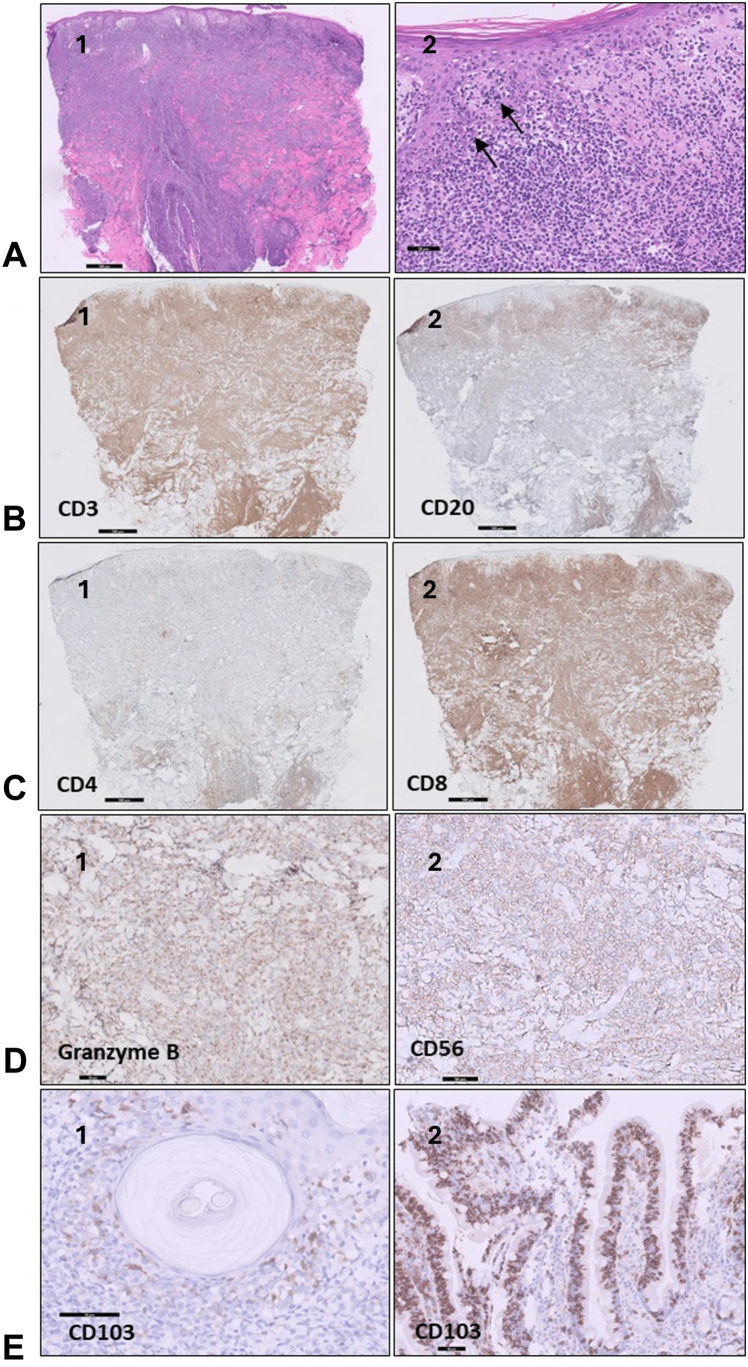
**A1,** Diffuse infiltrate throughout the dermis, with focal infiltrate of the epidermis and alignment along the dermal-epidermal junction (×10) **(A2,** arrows, ×20**)**. **B1,** The cells are positive for CD3 (×10), **(B2)** with an aberrant CD20 expression (×10). **C1** and **C2,** (×10), **(D1** and **D2)** (×20): Cells are positive for CD8, granzyme B, and CD56. **E,** CD103 staining is positive both in the skin **(E1)** and in the GI epithelium **(E2)** (×40)

Skin biopsies (Fig 2) showed nodular and diffuse lymphoid infiltrates composed of small and medium-sized atypical lymphocytes positive for CD3 and CD7, negative for CD5, CD30, and decreased CD2. The majority of the T-cells were CD8-positive (CD8/CD4 ratio ∼4-5). CD20 was focally positive; however, CD79, CD10, BCL6, BCL2, CD21, CD23, and MUM1 were negative. The proliferation marker Ki67 was approximately 70%. The atypical lymphocytes showed epitheliotropism, mainly in the basal layer. On additional immunostains, most atypical lymphocytes were positive for bF1, CD56, granzyme B, perforin, and TIA1, while EBV ISH was negative. Notably, CD103 was positive in the lymphocytes lining up the epidermis, as was seen in the duodenal biopsy, which was retrospectively revised. T-cell receptor (TCR) analysis showed identical monoclonal profile of T-cell population in the skin and in the deudenum.

According to the clinical and histological findings, the diagnosis of MEITL was made with cutaneous involvement as a presenting sign.

PET-CT scan revealed high FDG uptake in the skin lesions, a necrotic lung lesion, omental lesions, a large ileal mass with mesenteric fat infiltration, and ascites.

The patient began chemotherapy using the CHOP regimen (cyclophosphamide, doxorubicin, vincristine, prednisone). After the first chemotherapy cycle, he developed sharp abdominal pain and evidence of intestinal perforation. He underwent surgical resection with ileostomy. Biopsy of the resected intestine confirmed the diagnosis of MEITL, and the same T-cell clone and immunophenotype were identified in the GI biopsies, including the duodenal biopsy performed prior to the skin rash. PET-CT revealed improvement in cutaneous lesions; however, new hypermetabolic lung lesion appeared, confirmed as lymphoma. He underwent bone marrow transplantation. Subsequently, he was hospitalized with sepsis and died shortly after.

## Discussion

**Fig 3:**
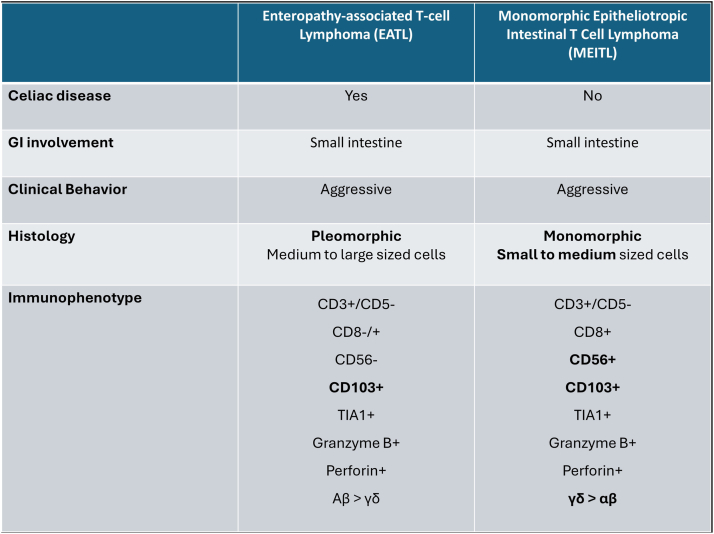
Differentiation between EATL and MEITL. *EATL*, Enteropathy-associated T-cell lymphoma; *MEITL*, monomorphic epitheliotropic intestinal T-cell lymphoma.

MEITL, formerly known as enteropathy-associated T-cell lymphoma (EATL) type II, is a rare and aggressive T-cell lymphoma originating from epithelial lymphocytes of the GI tract. Although MEITL is classified historically as a subtype of EATL, it is distinct in its lack of association with CD, complicating diagnosis (Fig 3).^2^, ^3^, ^4^

Symptoms of MEITL are nonspecific, including abdominal pain, diarrhea, and weight loss. About 50% of patients experience acute GI symptoms, including bleeding and perforation, often following chemotherapy, as observed in this case. Skin involvement is rarely reported. Delabie et al^5^ found cutaneous involvement in only 5% of 65 MEITL patients, with only 3 other case reports describing cutaneous manifestations like erythema multiforme-like eruptions, necrotic plaques, and solitary nodule.^6^, ^7^, ^8^ Our patient presented with a multifocal eruption composed of plaques (some crusted) and nodules, further emphasizing the cutaneous manifestation of this rare entity. More importantly, this case highlights that skin involvement can be the first clinical manifestation of this aggressive lymphoma.^6^

MEITL is not typically associated with CD, unlike EATL. However, 2 case reports have linked CD with MEITL.^3^ Our patient had a family history of CD, an association that may warrant further research.

Histologically, MEITL is characterized by monotonous infiltration of medium-sized lymphocytes expressing CD3 and CD8. A cytotoxic phenotype is typically present, with positive expressions of granzyme B, TIA-1, and perforin, as seen in this case.^2^^,^^4^^,^^6^ CD20 expression, reflecting an abnormal phenotype of the malignant T-cells, is variable (∼20%) and correlates with a worse prognosis.^9^ TCR clonality is confirmed by positivity for TCRβ and TCRγ, with TCRγ being more frequently observed. However, there is no established correlation between TCR clonality and clinical outcomes.^2^

Atypical immunophenotype of MEITL in addition to CD20 expression include lacking CD8 and CD56. Morphological features such as necrosis, angiotropism, and a starry-sky pattern have been described.^2^^,^^4^ Recent genomic studies have identified mutations in genes such as SETD2, STAT5B, JAK3, and TP53, contributing to the disease’s pathogenesis. These genetic alterations affect clinical outcomes, with less favorable prognosis seen with STAT5B and TP53 mutations, or MYC expression that was lacking in our patient.^2^, ^3^, ^4^

Therapeutic strategies for MEITL remain limited due to the lack of specific guidelines. The CHOP chemotherapy regimen and surgical interventions are the mainstay treatments, often providing suboptimal results. Emerging therapies, including pralatrexate and histone-deacetylase inhibitors, have shown promise.^4^^,^^10^ Autologous stem-cell transplantation may improve survival rates. However, the prognosis remains poor, with a disease progression-free survival of 1 month and an overall survival of 7 months, underscoring the need for more effective treatments.^1^

MEITL represents a complex entity with diverse clinical features, necessitating a multidisciplinary approach for accurate diagnosis and optimal management.

Dermatologists' and dermatopathologists' awareness of this aggressive lymphoma when cutaneous involvement is present, is crucial to initiate life-saving treatments to improve patient survival.

## Conflicts of interest

None disclosed.
